# Interact to Survive: *Phyllobacterium brassicacearum* Improves Arabidopsis Tolerance to Severe Water Deficit and Growth Recovery

**DOI:** 10.1371/journal.pone.0107607

**Published:** 2014-09-16

**Authors:** Justine Bresson, François Vasseur, Myriam Dauzat, Marc Labadie, Fabrice Varoquaux, Bruno Touraine, Denis Vile

**Affiliations:** 1 Laboratoire d′Ecophysiologie des Plantes sous Stress Environnementaux (LEPSE), UMR759, Institut National de la Recherche Agronomique-SupAgro, Montpellier, France; 2 Laboratoire des Symbioses Tropicales et Méditerranéennes (LSTM), UMR113, Université Montpellier 2-IRD-CIRAD-INRA-SupAgro, Montpellier, France; 3 Max Planck Institute for Developmental Biology, Tübingen, Germany; Institute of Genetics and Developmental Biology, Chinese Academy of Sciences, China

## Abstract

Mutualistic bacteria can alter plant phenotypes and confer new abilities to plants. Some plant growth-promoting rhizobacteria (PGPR) are known to improve both plant growth and tolerance to multiple stresses, including drought, but reports on their effects on plant survival under severe water deficits are scarce. We investigated the effect of *Phyllobacterium brassicacearum* STM196 strain, a PGPR isolated from the rhizosphere of oilseed rape, on survival, growth and physiological responses of *Arabidopsis thaliana* to severe water deficits combining destructive and non-destructive high-throughput phenotyping. Soil inoculation with STM196 greatly increased the survival rate of *A. thaliana* under several scenarios of severe water deficit. Photosystem II efficiency, assessed at the whole-plant level by high-throughput fluorescence imaging (*F*_v_/*F*_m_), was related to the probability of survival and revealed that STM196 delayed plant mortality. Inoculated surviving plants tolerated more damages to the photosynthetic tissues through a delayed dehydration and a better tolerance to low water status. Importantly, STM196 allowed a better recovery of plant growth after rewatering and stressed plants reached a similar biomass at flowering than non-stressed plants. Our results highlight the importance of plant-bacteria interactions in plant responses to severe drought and provide a new avenue of investigations to improve drought tolerance in agriculture.

## Introduction

Drought is a global concern and episodes of severe drought will most probably be more frequent with dramatic consequences on agriculture [1]. Severe water stress greatly reduces plant biomass production and can lead to plant mortality [2]. Over the last decade, it has been shown that plants can largely benefit from their interactions with soil microorganisms; especially with plant growth promoting rhizobacteria (PGPR) that colonize the rhizosphere of many plants species [3]. The stimulation of growth by PGPR is often associated with lower plant susceptibility to various biotic and abiotic stresses [4], [5] and there is a growing interest in the use of these rhizobacteria in agriculture [6], [7].

Survival to drought events is found in plants that are able to maintain key cellular functions under severe water stress and recover similar pre-stress values when conditions become favorable again [8]. The capacity to tolerate low leaf water status, or dehydration tolerance, is widely variable among species [9]. The most spectacular adaptation to severe drought is illustrated by resurrection plants [10]. These plants display rapid physiological responses and metabolic adjustments [11], and tolerate nearly complete tissue dehydration. During mild drought or water stress of limited duration, plants that maintain a good water status can complete their life cycle, although often with reduced performance. However, when stress becomes more drastic or is prolonged the leaf water potential drops and leaf damages occur [12]. Then, dramatic reduction of biomass production and even plant mortality appear [2]. To prevent tissue damages, and survive at low leaf water content, many processes and signaling pathways are involved [13]. Osmotic adjustments and accumulation of specific protective osmolytes such as proline [14], glycine betaine [15] or trehalose [16] allow stabilizing cellular structures. One of the most rapid responses to prevent hydraulic failure is stomatal closure. However under severe water stress, stomatal closure can diminish photosynthetic uptake and induce carbon starvation [17] that can lead to total or partial leaf senescence. Drought-induced senescence of older leaves can contribute to water saving, while allowing the reallocation of nutrient to the younger leaves [18]. However, leaf senescence alters photosynthetic functioning and chlorophyll (Chl) properties [19]. Chl-fluorescence is a powerful, rapid and minimally invasive indicator of plant health [20]. In particular, dark-adapted measurements of the ratio of variable to maximal fluorescence (*F*_v_/*F*_m_) give the potential quantum yield (or efficiency) of the photosystem II (PSII) photochemistry, which varies with plant water status [21], [22]. Decrease in *F*_v_/*F*_m_ is due to an increase in leaf damages that may to some extent be reversible [22]. After a period of water stress, it has been shown that plants have the capacity to recover progressively, but sometimes incompletely, their photosynthetic [23], [24] and growth [25] potential. During stress, plant growth rate is reduced, even stopped, but leaf cells retain their ability to expand when conditions become favorable again [25].

Rhizobacteria can help plants to cope with negative effects of water deficit. Under water stresses of moderate intensity, some PGPR can improve resistance to water deficit through i) modifications in phytohormones content and/or signaling, notably ethylene, auxin, cytokinin, and abscisic acid (*e.g.*, [26], [27], [28], [29]), ii) enhanced cells detoxification by increasing antioxydase activities such as catalase [30] or superoxide dismutase [31], iii) changes in plant functional traits such as photosynthetic capacity through changes in chlorophyll content [31] and in photosynthetic PSII efficiency [32], [33], or iv) the formation of a biofilm which enhances soil aggregation and improves water stability in the soil [34]. Even though a rich literature exists on plant responses to rhizobacteria under water stress (for reviews see [4], [35]), studies of PGPR effects on plant survival are surprisingly limited.

The aim of this study was to investigate the effects of the free-living PGPR, *Phyllobacterium brassicacearum* strain STM196, on survival, growth and physiological responses of *A. thaliana* during the time-course of severe drought progression. The STM196 strain belongs to the *Phyllobacteriaceae* family in the *Rhizobiales*, order of α-*Proteobacteria* [36]. This strain was the most efficient PGPR isolated from the rhizoplan of field-grown *Brassica napus* roots [37], [38]. We have recently shown that STM196 improves *A. thaliana* resistance to moderate water deficit through a reproductive delay and changes in transpiration rate correlated to modifications of leaf ABA content [29]. Moreover, previous *in vitro* studies showed that STM196 modifies root architecture and hormonal signaling [39], [40], [41], [42]. Here, our main experimental goals were (*i*) to determine whether plant-PGPR interaction mitigate the negative consequences of severe drought on plant survival, (*ii*) to assess how biotic interactions with PGPR influence physiological mechanisms of plants (*iii*) to evaluate the benefits of inoculation on growth and productivity of plants after stress. *A. thaliana* plants were subjected to five scenarios of severe soil water deficit, with progressive soil drying and rewatering treatments. The use of the plant phenotyping platform PHENOPSIS allowed fine-tuning of soil water content and daily acquisition of images of plants [43]. The dynamics of physiological changes in plants were investigated independently in surviving and perishing plants under severe drought by estimating survival with non-invasive chlorophyll fluorescence measurements at high throughput levels. This approach is broadly applicable to investigate survival of plants under various stresses affecting chlorophyll properties and leaf functioning.

## Materials and Methods

### Bacteria material, bacterial inoculum and soil inoculation

The *Phyllobacterium brassicacearum* STM196 strain was grown for three days in Petri dishes on a sterile (20 min at 120°C) 1.5% agar (w/v; Sigma-Aldrich) medium (E′) containing 2.87 mM K_2_HPO_4_, 0.81 mM MgSO_4_, 1.71 mM NaCl, 7.91 mM KNO_3_, 0.34 mM CaCl_2_, 30 µM FeCl_3_, 1% mannitol (w/v) and 0.3% yeast extract (w/v; Sigma-Aldrich), adjusted to pH 6.8. Next, the bacteria were grown aerobically in liquid E′ medium on a rotary shaker (145 rpm) at 25°C for 24 h to reach the exponential phase of growth. Culture of bacteria cells was pelleted by centrifugation (3200 g, 15 min, 20°C) and resuspended in deionized water. To obtain 3.10^7^ colony forming units (cfu) per gram of soil, the volume was adjusted based upon a correspondence with the absorbance measured at 595 nm (WPA UV 1101, Biotech Photometer, Cambridge, UK). This inoculum was directly put into the non-sterilized soil substrate (see Table S1 in File S1 for soil chemical properties), which was then manually homogenized.

### Plant material, growth conditions and irrigation treatments

**Figure 1:**
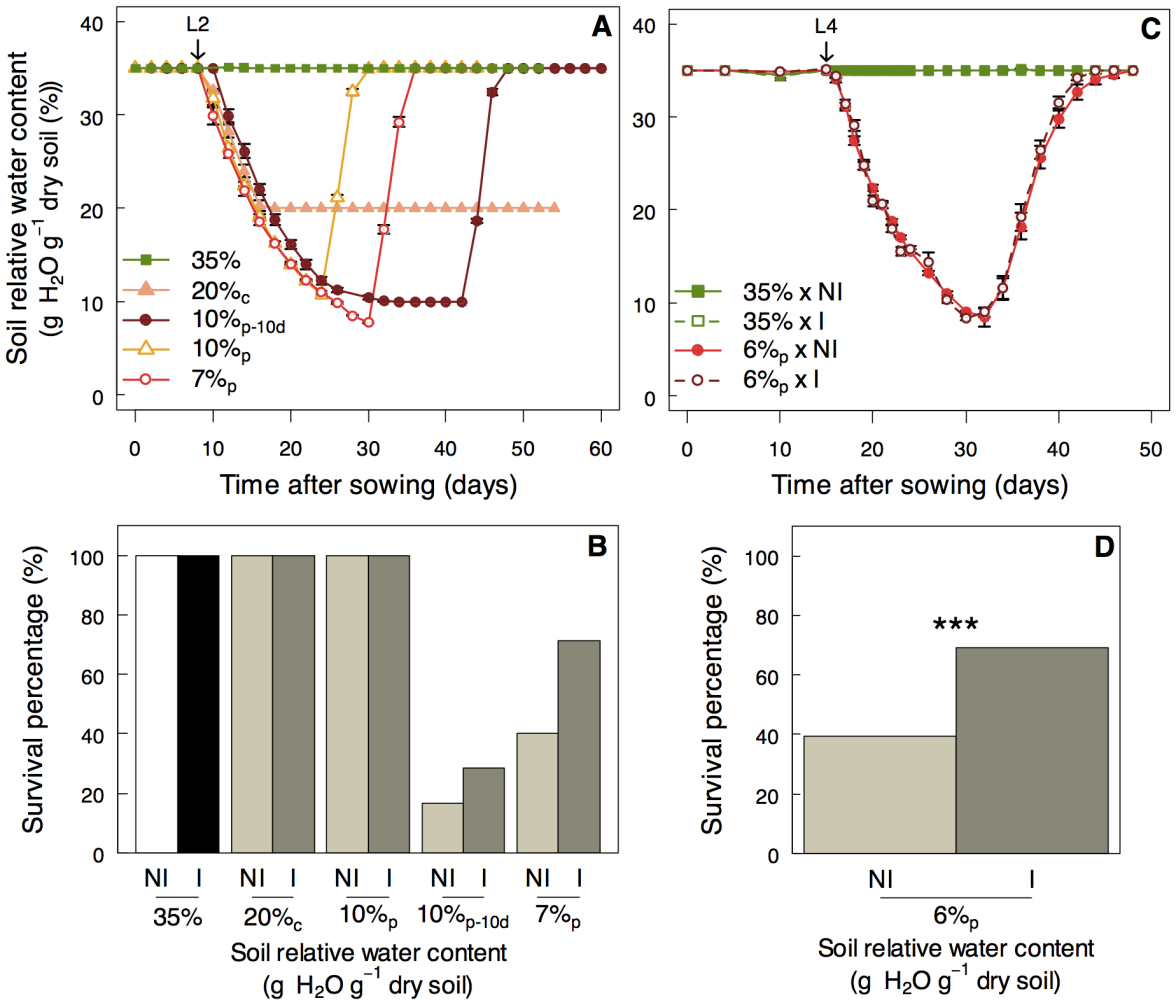
*Phyllobacterium brassicacearum* STM196 enhances *A. thaliana* survival under severe water deficits. **A)** Average soil relative water content and **B)** survival percentage of non-inoculated (NI) and inoculated plants (I) in five watering scenarios including constant well-watered conditions (35% g H_2_O^−1^ dry soil; 35%), water withdrawing from the two firsts leaves (L2) followed by constant moderate water deficit (20% g H_2_O^−1^ dry soil; 20%_c_), punctual severe water deficits with rewatering after 10 days at 10% g H_2_O^−1^ dry soil (10% g H_2_O^−1^ dry soil; 10%_p-10d_) or after1 day (10%_p_), and after 1 day at 7% g H_2_O^−1^ dry soil (7%_p_). **C)** Average soil relative water content and **D)** survival percentage of NI (closed symbols) and I (open symbols) plants in two watering scenarios including constant well-watered conditions (35% g H_2_O^−1^ dry soil; 35%), and water withdrawing from the four-leaves stage (L4) followed by rewatering after 1 day at 6% g H_2_O^−1^ dry soil (6%_p_). Asterisks indicate significant differences following Chi^2^ test between NI and I plants (***: *P*<0.001).

All experiments were realized with *A. thaliana* (L.) Heynh accession Col-0. Five seeds were sown at the soil surface in 260 mL culture pots filled with a damped mixture (1∶1, v:v) of loamy soil and organic compost (Neuhaus N2; see Table S1 in File S1 for soil chemical properties) inoculated with STM196 or not. Non-inoculated soil was previously damped with deionized water to avoid difference in initial soil humidity with inoculated soil. Soil water content was controlled during pot filling by determining soil fresh weight (FW_soil_) and soil dry weight (DW_soil_, after 5 d at 60°C) every ten pots. Initial soil relative water content was determined as RWC_soil_  =  (FW_soil_ – DW_soil_)×100×DW_soil_^−1^. The pots were kept in the dark for two days in the PHENOPSIS growth chamber [43] and were damped with sprayed deionized water three times a day until germination. Then, plants were cultivated under 12 h day length (180 µmol m^−2^ s^−1^ photosynthetic photon flux density, PPFD, at plant height). During germination phase (7 d), air temperature was set to 20°C day and night, and air relative humidity was adjusted in order to maintain constant water vapor pressure deficit (VPD) at 0.6 kPa. Then, plants were grown at 20/17°C day/night and 0.8 kPa of VPD. Seedlings with similar sizes and developmental stages were selected and thinned to one to four plants per pot just before the beginning of water stress (see Figure 1A, C and Table 1, for watering scenarios and details on replicate numbers). Soil water content was daily adjusted with a modified one-tenth-strength Hoagland solution [44]. Soil water content was maintained at 0.35 g H_2_O g^−1^ dry soil in the well-watered treatment (35%, WW) and it was decreased progressively to the desired RWC_soil_ by stopping irrigation in the water deficit treatments (WD; Table 1). Continuous moderate water deficit (20%_c_) was maintained at 0.20 g H_2_O g^−1^ dry soil during the whole plant life cycle. In the case of severe punctual stresses, when the soil reached the desired RWC_soil_ level depending on the experiment (*i.e.*, 0.10, 0.07 or 0.06 g H_2_O g^−1^ dry soil), irrigation was resumed after 1 day (for 10%_p_, 7%_p_ and 6%_ p_ stresses) or after 10 days (for 10%_p-10d_) to progressively reach the WW soil condition (avoiding no more than 10 ml of the modified Hoagland solution per day to avoid soil leaching). Soil water content was then maintained at WW until final harvests at first flower open (stage 6.00; [45]).

**Table 1:** Description of watering scenarios and design of experiments.

			Water stress characteristics				Experiment		
Treatment	Watering scenario	Soil water potential (Mpa)	Stage of irrigation withdrawing	Days to reach desired RWC_soil_	Days before rewatering	Number of plants	1	2	3
35%_c_; WW	RWC_soil_ maintained at 35% by daily irrigation during the whole plant life cycle	0.07	No stress			13–16	x	x	x
20%_c_	Irrigation stopped at stage L2 to decrease RWC_soil_ at 20% and RWC_soil_ thereafter maintained constant during the whole plant life cycle	−0.28	L2	6	No rewatering	9–20		x	
10%_c-10d_	Irrigation stopped at stage L2 to decrease RWC_soil_ at 10%. RWC_soil_ then maintained constant during 10 days before rewatering to reach well-watered level (RWC_soil_ = 35%)	−3.19	L2	15	10	7–12	x		
10%_p_	Irrigation stopped at stage L2 to decrease RWC_soil_ at 10%. RWC_soil_ then maintained constant during 1 day before rewatering to reach well-watered level	−3.19	L2	14	1	7–12	x		
7%_p_	Irrigation stopped at stage L2 to decrease RWC_soil_ at 7%. RWC_soil_ then maintained constant during 1 day before rewatering to reach well-watered level	−9.52	L2	20	1	7–10	x	x	
6%_p_	Irrigation stopped at stage L4 to decrease RWC_soil_ at 6%. RWC_soil_ then maintained constant during 1 day before rewatering to reach well-watered level	_	L4	30	1	48–52			x

WW: well-watered condition (control); RWC_soil_: soil relative water content (%; g H_2_O g^−1^ dry soil); L2: emergence of the first two leaves (stage 1.02; [45]) and L4: emergence of the four-leaf stage (stage 1.04; [45]).

All treatments were performed in soil inoculated or not with *Phyllobacterium brassicacearum* STM196. See Figure 1A, C for a graphical representation of watering scenarios.

Soil water potential was determined during soil drying (from 0.35 to 0.06 g H_2_O g^−1^ dry soil, Table 1 and Figure S1 in File S1; WP4-T dewpoint meter, Decagon Devices, Pullman, WA 99163, USA).

### Plant survival

Plants that failed to develop after rewatering and deteriorated until the complete senescence of the rosette were considered as dead. Survival percentage was scored in three consecutive experiments that were carried out following the same experimental procedure (Table 1). In experiments 1 and 2, water stresses (20%_c_, 10%_p-10d_, 10%_p_ and 7%_p_) were started at the emergence of the first two leaves (L2; stage 1.02; [45]). In experiment 3, the number of replicates was increased in order to maximize the statistical power, water stress was started at four-leaf stage (L4; stage 1.04; [45]) to allow precise measurements on early developmental stages and RWC_soil_ was decreased to reach 0.06 g H_2_O g^−1^ dry soil (6%_p_ stress) before rewatering to reach well-watered soil condition (RWC_soil_ = 35%).

### Measurements of whole-plant traits

Detailed plant phenotyping of growth and physiological status was performed in experiment 3 (6%_p_ stress) throughout the whole plant cycle.

#### Measurement of photosynthetic efficiency

Measurement of photosynthetic efficiency was daily performed from early developmental stages to the emergence of the flowering stem (*i.e.*, bolting stage) under WW and water deficit. The maximum quantum yield of PSII was estimated by the ratio of variable to maximal chlorophyll fluorescence (*F*_v_/*F*_m_) on dark-adapted plants, after 8–12 h of dark (IMAGING-PAM; Maxi-version; W-IMAG-K6 camera implemented in PHENOPSIS; Imaging Win software; Walz; Effeltrich, Germany). *F*_v_/*F*_m_ is given by (*F*_m_-*F*_0_)/*F*_m_ [46], where *F*_0_ is the basal fluorescence in the dark adapted state and *F*_m_ is the maximal fluorescence obtained after saturating light pulse (Si 9, width 800 ms). For unstressed plants, the value of *F*_v_/*F*_m_ around 0.83 measured for most plant species and values lower than this indicate that plants are stressed [46]. Whole-rosette *F*_v_/*F*_m_ values were extracted by image analyses using ImageJ (ImageJ 1.47V, Rasband, Bethesda, Maryland, USA).

#### Determination of plant water status

To determinate plant relative water content (RWC_leaf_), plants were harvested at different levels of RWC_soil_ during establishment of water stress (*i.e.*, at 0.35, 0.20, 0.10 and 0.06 g H_2_O g^−1^ dry soil) and after rewatering (*i.e.*, at 0.20r, 0.10r and 0.35r g H_2_O g^−1^ dry soil and at first flower open). Rosettes were cut and immediately weighted, after the removal of inflorescence stems for plants harvested at stage 6.00 [45], to determine aboveground vegetative fresh mass (FM). The rosettes were wrapped in moist paper and placed into Petri dishes at 4°C in darkness overnight to achieve complete rehydration. Water-saturated fresh mass (SM) was then determined. The rosettes were oven-dried at 65°C for 48 h, and rosette dry mass (DM) was determined. From these measurements, relative water content (RWC_leaf_ = (FM – DM)×100×(SM – DM)^−1^) was calculated at the rosette level. Water content (WC_leaf_) was calculated as FM × DM^−1^ ratio.

#### Rosette expansion during time course

Projected area of the rosettes (RA*_proj_*) was determined every days from semi-automated analysis (ImageJ 1.43C [47]) of zenithal images of the plants (Sony SSC-DC393P camera). A sigmoid curve was fitted for each plant following RA_proj_ = *a*/[1+exp−[(*d*−*a*/2)/*b*]] where *a* is the maximum area, and *d* is the number of days after sowing. The maximum rate of leaf expansion (R_max_, mm^2^ d^−1^) was calculated from the first derivative of this logistic model at *d_0_* as R_max_ = *a*/(4*b*).

Flowering time was determined as the number of days from germination until visualization of the first flower open.

#### Measurements of leaf morphology at flowering

Surviving individuals were harvested at first flower open. Rosettes were cut and immediately weighted after the removal of inflorescence stems to determine aboveground vegetative FM. SM was then determined as describe above. Total leaf number was determined, and the leaf blades were separated from their petiole in order of leaf emergence and scanned for measurements of individual leaf area (ImageJ 1.43C). Leaf blades, petioles and reproductive structures were then separately oven-dried at 65°C for 48 h, and their dry mass was determined. Rosette DM was calculated as the sum of blades and petioles dry masses and RWC_leaf_ was calculated at the rosette level. All phenotypic data were stored in the PHENOPSIS database [47].

### Quantification of bacteria in the soil

To analyze bacterial growth under water stress in soil, a natural mutant of STM196 strain was selected in a selection medium E′ containing 100 µg ml^−1^ of rifampin and then, was transformed using pCH60 vector. The vector pCH60 encodes for tetracycline resistance and contains the *gfp* gene that is constitutively expressed [48]. Bacterial concentration was estimated during soil drying at 0.35, 0.20, 0.10 and 0.06 g H_2_O g^−1^ dry soil and after rewatering at 0.35r g H_2_O g^−1^ dry soil. Quantification of bacteria was performed in soil without plant. The concentration of colony-forming units (cfu/mg) was estimated using the most probable number method (MPN; [49]). 100 mg of inoculated soil were put in 1 ml of physiological water (8.5 g l^−1^ de NaCl) on a rotary shaker (145 rpm) at 25°C for 2∶30. The solubilized soil samples were serially diluted until 10^−7^, and 100 µl were spread in Petri dishes on a sterile (20 min at 120°C) 1.5% agar (w/v; Sigma-Aldrich) medium (E′) with addition of 50 µl of rifampin and tetracycline. Bacteria were then counted after 6 days at 25°C.

### Statistical analyses and determination of the lethal *F*_v_/*F*_m_ threshold

All analyses were performed using R 2.15 [50]. Comparisons of mean trait values between treatments were performed with Kruskal–Wallis non-parametric tests. Plant survival was analyzed by Chi^2^ tests. To estimate the survival of harvested plants during water stress (only for 6_p_% stress), a 90% lethal threshold was determined just before rewatering (*i.e.*, at 0.06 g H_2_O g^−1^ dry soil) from plants with known survival, in a dose-response analysis of survival as a function of *F*_v_/*F*_m_ values. The relationships between survival probability and whole-rosette *F*_v_/*F*_m_ values were modeled using a binomial logistic regression. The effect of inoculation was tested by Chi^2^ tests on deviance ratio. The 90%-mortality threshold (*i.e.*, 10% survival probability) of *F*_v_/*F*_m_ value was inferred from the regression. Plants with *F*_v_/*F*_m_ values above this threshold were considered as able to survive the stress imposed whereas plants with *F*_v_/*F*_m_ values below this threshold were considered as perishing plants. Estimated mortality ratios (*i.e.*, proportion of perishing plants) were compared by Chi^2^ tests.

## Results

### *Phyllobacterium brassicacearum* STM196 strain increases *A. thaliana* survival under multiple scenarios of severe water deficit

*Arabidopsis thaliana* Col-0 was grown under five scenarios of soil water availability to determine a level of stress that induced plant mortality and then analyze the effects of STM196 strain on plant survival. Soil relative water content was maintained at 0.35 g H_2_O g^−1^ dry soil in the well-watered (WW) treatment until flowering and it was decreased progressively to the desired RWC_soil_ by stopping irrigation in the water deficit treatments followed by rewatering or not (see Table 1 and Figure 1A, C for a description of the watering treatments). Under WW conditions, all plants survived and reached the reproductive stage (Figure 1A, B). All plants also survived a continuous moderate WD (20%_c_; Figure 1A, B), *i.e.* irrigation withdrawn from two first leaves emerged (L2) and RWC_soil_ then maintained at 20% g H_2_O g^−1^ dry soil until flowering. Decreasing RWC_soil_ punctually to 10% g H_2_O g^−1^ dry soil (10%_p_) did not affect plant survival, but when this RWC_soil_ level was prolonged for 10 days (10%_p-10d_) more than 80% of the non-inoculated plants died (Figure 1A, B). Decreasing RWC_soil_ punctually to 7% g H_2_O g^−1^ dry soil (7%_p_) resulted in 40% of non-inoculated plants that survived and reproduced after stepwise rewatering to WW conditions (Figure 1A, B).

To perform accurate measurements of plant development and physiology during soil drying, the beginning of water stress was delayed to four leaves emerged (L4), and RWC_soil_ was punctually decreased to 6% (6%_p_; Figure 1C). Under this scenario, plant survival rate of non-inoculated plants was 40%, *i.e.* similar to the rate observed under punctual 7%_p_ stress (Figure 1B, D). In all watering scenarios causing plant mortality (10%_p-10d_, 7%_p_ and 6%_p_), soil inoculation by STM196 strain resulted in a great increase in plant survival rate (Figure 1B, D). For instance, 70% of inoculated plants survived against only 40% of non-inoculated plants under 6%_p_ stress (*P*<0.001). This stress level was reached 1.7 days earlier in inoculated plants than in non-inoculated plants (the mean ± SE number of days to reach 6% RWC_soil_ was 16.8±1.9 (*n* = 50) and 18.5±2.2 (*n* = 48) for inoculated and non-inoculated plants, respectively; *P*<0.001). To decipher the effects of STM196 (only under 6%_p_ stress) at similar RWC_soil_ levels, the traits of stressed plants were analyzed and presented independently of time but as a function of soil humidity during soil drying and after rewatering. The growth of STM196 in the soil was also analyzed during the WD treatment, without plant. Bacterial growth was not affected by WD and the concentration of bacteria remained constant during the experiment (Figure S2 in File S1).

### STM196 strain delays and reduces plant mortality under severe water deficit

**Figure 2:**
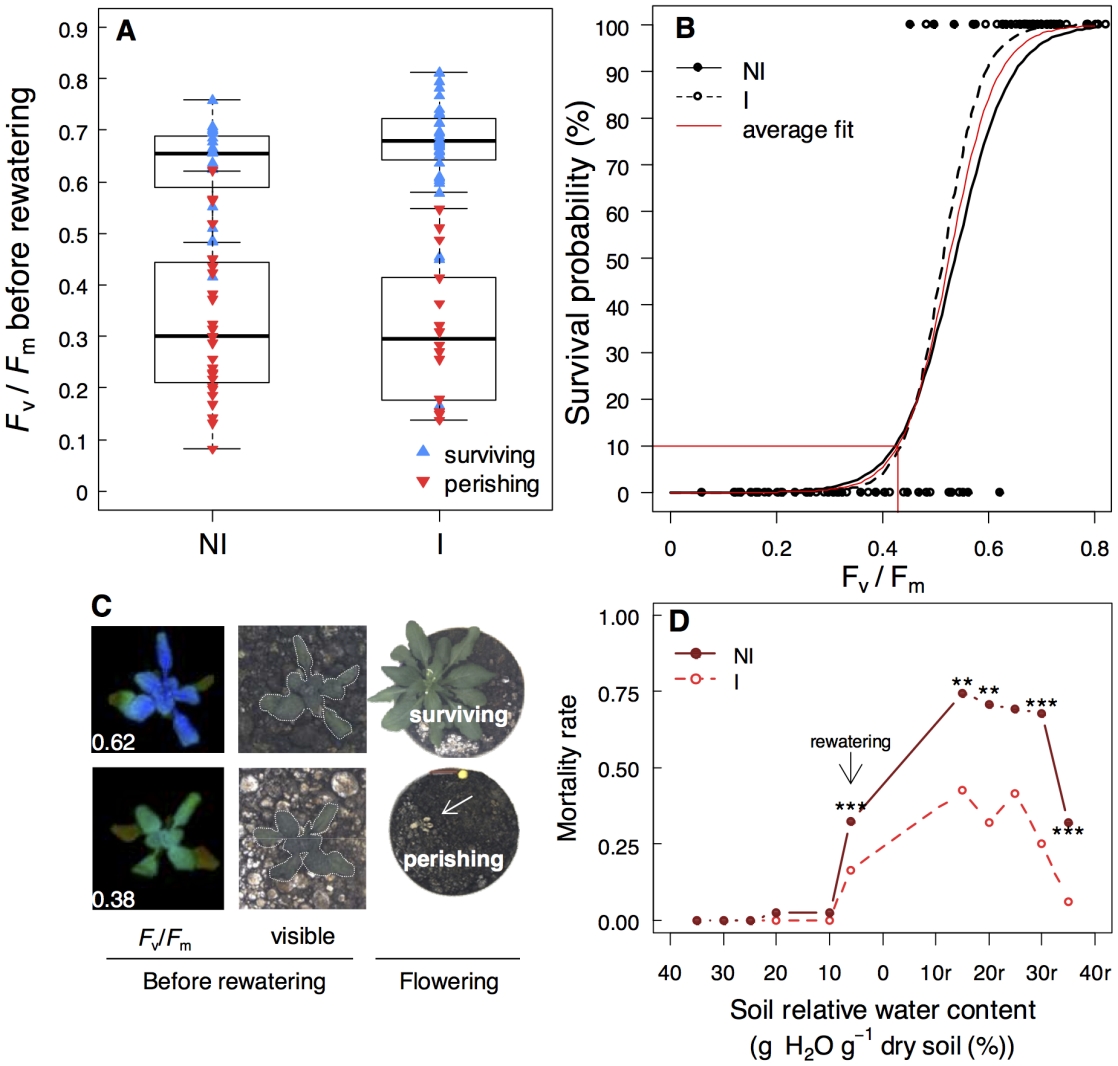
*P. brassicacearum* STM196 does not affect *A. thaliana* mortality threshold but delays and reduces mortality rate during soil drying. **A)** Whole-rosette *F*_v_/*F*_m_ just before rewatering (*i.e.*, 0.06 g H_2_0^−1^ dry soil) of non-inoculated (NI) and inoculated (I), and surviving (▴; n = 19–36) and perishing (▾; n = 16–29) plants as observed at the end of the experiment conducted with water withdrawing followed by rewatering at 6% g H_2_O^−1^ dry soil (6%_p_ stress in Figure 1). **B)** Relationships between *F*_v_/*F*_m_ and survival probability (same data as in A); the 90%-mortality threshold (*F*_v_/*F*_m_ = 0.398) is shown. **C)** *F*_v_/*F*_m_ false-colour images (left) and visible images (middle) of vegetative rosettes before rewatering and of surviving flowering and perishing plant at the end of the experiment (right). **D)** Mortality rate of stressed NI (closed symbols) and I (open symbols) plants during soil drying and rewatering, as estimated from the 90%-mortality threshold. Asterisks indicate significant differences following Chi^2^ test between NI (n = 28–242) and I (n = 16–187) plants (**: *P*<0.01; ***: *P*<0.001).

Non-destructive measurements of Chl-fluorescence were used as a sensitive indicator of photosynthetic performance (efficiency of PSII) from early developmental stages to the emergence of flowering stem. Under WW conditions, whole-rosette mean *F*_v_/*F*_m_ was 0.80 during the entire life cycle and was not affected by soil inoculation with STM196 (*P* = 0.57; see Figure S3 in File S1). As expected, *F*_v_/*F*_m_ decreased significantly under severe WD (6_p_% stress). Mean *F*_v_/*F*_m_ just before rewatering (*i.e.*, RWC_soil_ = 6% g H_2_O g^−1^ dry soil) was equal to 0.7 for surviving plants whereas it was equal to 0.3 for the plants that failed to develop and perished after rewatering, for both non-inoculated and inoculated plants (Figure 2A, C). A 90% lethal threshold was then determined with *F*_v_/*F*_m_ values of these latter plants in order to estimate the mortality of harvested plants with unknown survival (Figure 2A, B). There was no difference between the logistic regressions of survival on *F*_v_/*F*_m_ performed on non-inoculated and inoculated plants (*P* = 0.518; Figure 2B) and the average fit was therefore used. The 90%-mortality threshold was inferred at *F*_v_/*F*_m_ = 0.398. In further analyses, plants with *F*_v_/*F*_m_ values above this threshold were considered as able to survive the stress imposed and plants with *F*_v_/*F*_m_ values below this threshold were considered as perishing. The distinction between surviving and perishing plants was crucial to avoid errors of interpretation of the results due to a higher number of inoculated surviving plants, and could help to differentiate the behavior of plants according to their ability to survive to WD. This threshold showed that estimated mortality rate (*i.e.*, the proportion of perishing plants) tended to increase at 20% g H_2_O g^−1^ dry soil in non-inoculated plants and never before 6% g H_2_O g^−1^ dry soil in inoculated plants (Figure 2D). From RWC_soil_ = 6% g H_2_O g^−1^ dry soil and after rewatering, the estimated mortality rate of inoculated plants was significantly lower than that of non-inoculated plants (Figure 2D; note that at the end of the experiment most senescing plants were no more detectable because decomposition started, which explains the biased decrease of mortality rate observed).

### Delayed dehydration of tissues confers a higher tolerance to photosynthetic damages in STM196-inoculated plants

**Figure 3:**
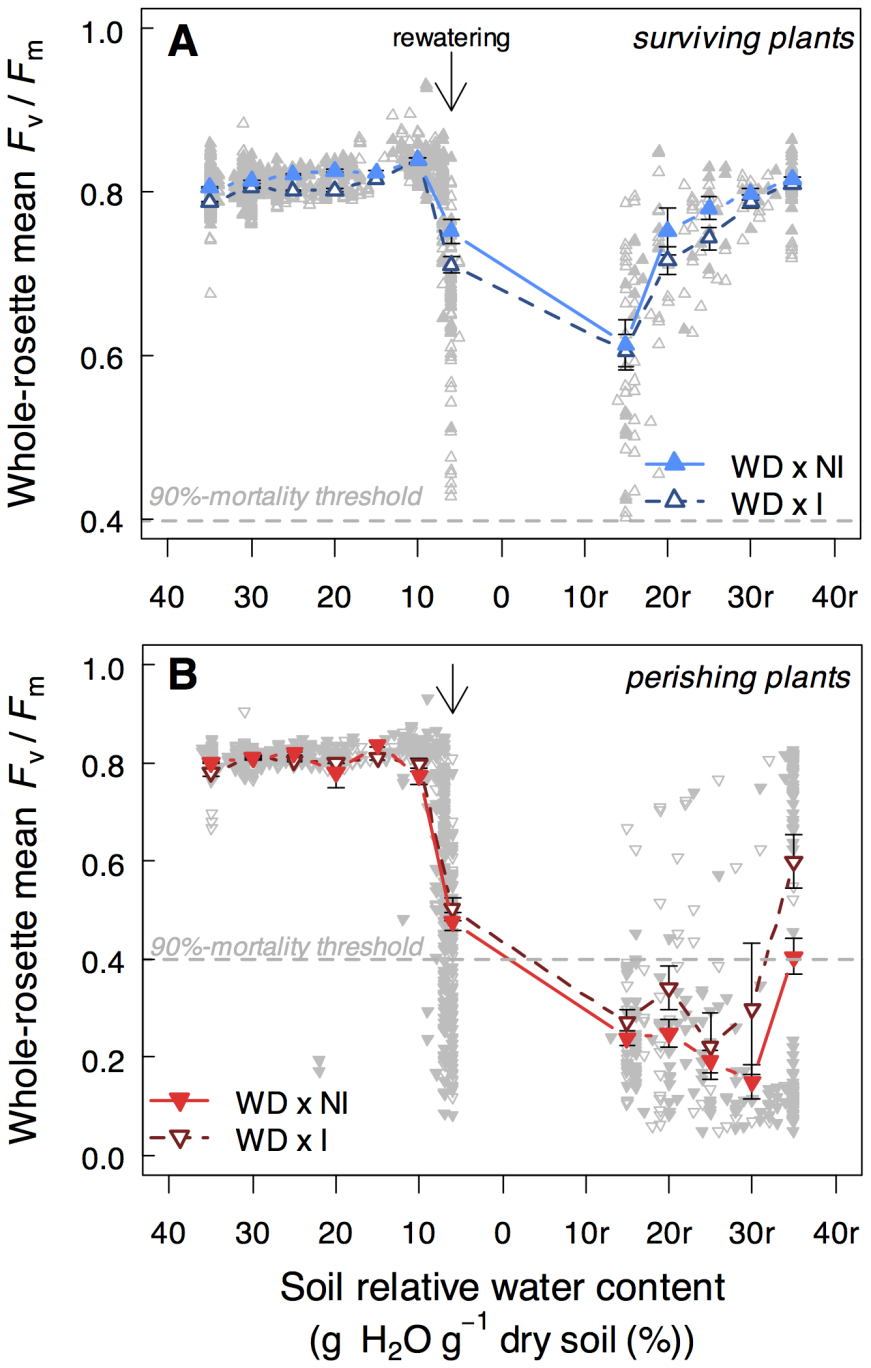
*P. brassicacearum* STM196 improves *A. thaliana* tolerance to higher levels of leaf photosynthetic damages under severe water deficit (WD; 6%_p_). Whole-rosette *F*_v_/*F*_m_ of **A)** surviving and **B)** perishing non-inoculated (NI; n_surviving_ = 7–147; n_perishing_ = 6–137) and inoculated (I; n_surviving_ = 10–152; n_perishing_ = 6–78) plants during soil drying and during rewatering. Dashed lines show the 90%-mortality threshold and arrows indicate the beginning of soil rewatering. Grey points represent individuals for each condition (NI; closed symbols and I; open symbols).

Whole-rosette Chl-fluorescence was then analyzed independently in surviving and perishing plants inoculated or not with STM196. At the whole-rosette level, the decrease in mean *F*_v_/*F*_m_ was not progressive in plants exposed to stress but was dramatically affected beyond 10% RWC_soil_ in both surviving and perishing plants, with a higher magnitude for the latter (Figure 3A, B). At the maximum of stress severity (*i.e.*, 6% RWC_soil_), lowering of whole-rosette mean *F*_v_/*F*_m_ was more pronounced in surviving inoculated plants than in non-inoculated plants (*P*<0.05), and *F*_v_/*F*_m_ of inoculated plants was closer to the mortality threshold (see grey points and dashed line in Figure 3A). Upon rewatering, whole-rosette mean *F*_v_/*F*_m_ of both non-inoculated and inoculated surviving plants recovered progressively *F*_v_/*F*_m_ values to reach initial mean *F*_v_/*F*_m_ (0.8), similar to non-stressed plants (Figure 3A and Figure S3 in File S1). Both inoculated and non-inoculated perishing plants reached an equivalent mean *F*_v_/*F*_m_ (0.49) at 6% RWC_soil_ (Figure 3B). This result suggests that inoculation by STM196 induced a slight decrease in photosynthetic performance but surviving inoculated plants had higher tolerance to photosynthetic damages under WD.

**Figure 4:**
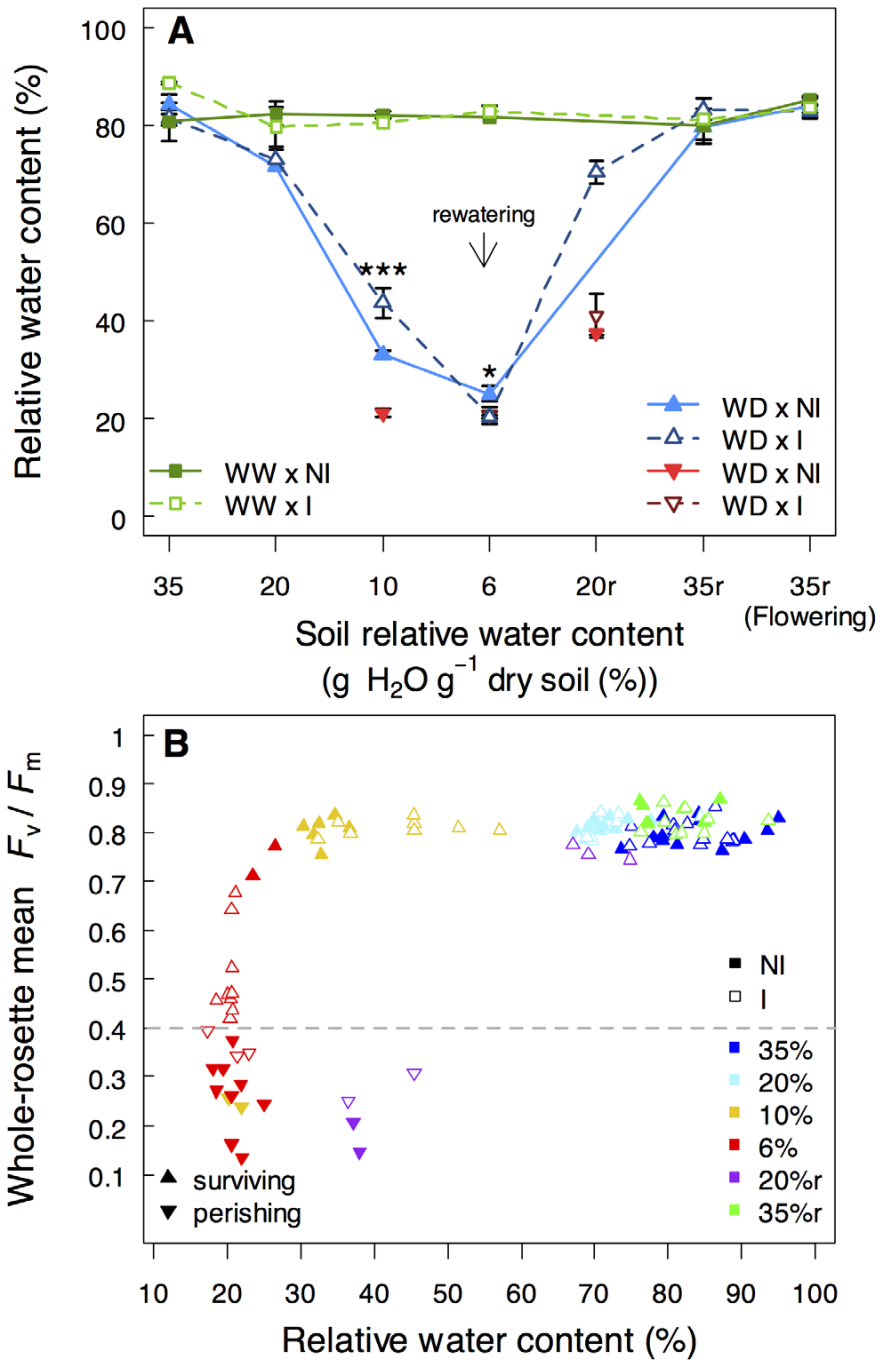
*P. brassicacearum* STM196 induces a delayed dehydration of tissues and increases tolerance to severe water deficit. **A)** Leaf relative water content and **B)** relationship between whole-rosette *F*_v_/*F*_m_ and leaf relative water content of non-inoculated (NI; closed symbols) and STM196-inoculated (I; open symbols) plants under well watered (WW) and water deficit (WD; 6%_p_) during soil drying (35%, 20%, 10% and 6%) and after rewatering (20%r, 35%r and 35%r at flowering). Arrow in A indicates the beginning of soil rewatering. Dashed line in B represents the 90%-mortality threshold. Surviving plants with mean *F*_v_/*F*_m_ values above the threshold, are represented by triangles (▴; n = 3–10 and n = 3–19 for NI and I plants, respectively) and perishing plants, with mean *F*_v_/*F*_m_ below the threshold, are represented by upside-down triangles (▾; n = 3–9 and n = 3 for NI and I plants, respectively). Asterisks indicate significant differences following Kruskal-Wallis tests between NI and I plants (*: *P*<0.05 and ***: *P*<0.001).

**Figure 5:**
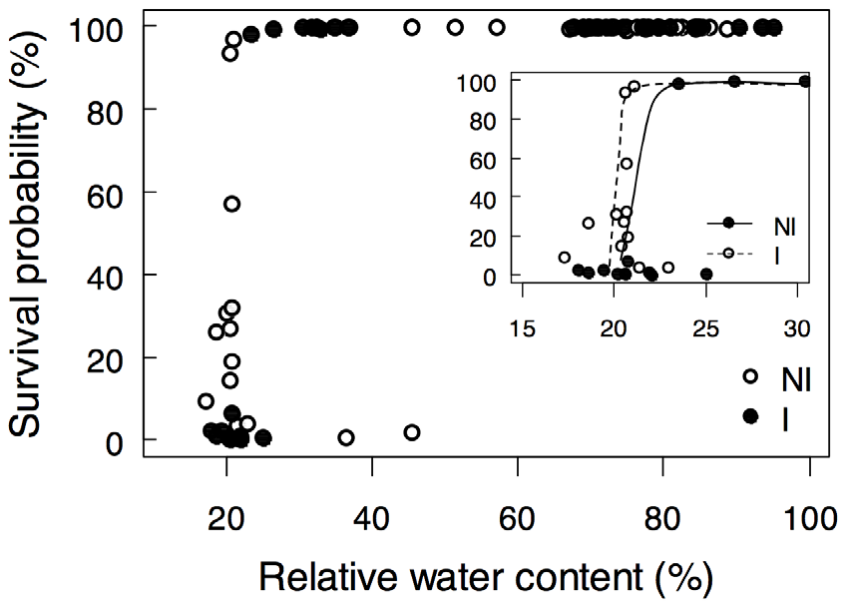
*P. brassicacearum* STM196 increases plant survival to severe leaf dehydration. Relationship between plant survival (estimated from whole-rosette *F*_v_/*F*_m_ values; Figure 1B) and leaf relative water content of non-inoculated (NI; closed circle; n = 36) and STM196-inoculated (I; open circle; n = 44) under severe water deficit (6%_p_). Insert represents fitting of logistic regression at very low leaf relative water content (solid and dashed lines for NI and I plants, respectively).

Severe WD in the soil unequivocally led to reduced water content in plant tissues (Figure 4A). RWC_leaf_ was progressively affected by soil drying and 6% RWC_soil_ resulted in a great decrease causing a RWC_leaf_ as low as 25% in non-inoculated plants compared to 82% in plants grown under WW conditions (Figure 4A). At 10% RWC_soil_, surviving inoculated plants displayed higher RWC_leaf_ (*P*<0.001) than non-inoculated plants, which suggested that soil inoculation by STM196 slowed the loss of water in the leaves. At 6% RWC_soil_ the effect of inoculation was opposite and the RWC_leaf_ of surviving inoculated plants was lower than that of non-inoculated plants (*P*<0.05). This result showed that soil inoculation by STM196 allowed plants to withstand higher leaf dehydration than non-inoculated plants. RWC_leaf_ and Chl-fluorescence were closely related (Figure 4B). The relationship between *F*_v_/*F*_m_ and RWC_leaf_ showed that inoculated plants displayed a lower decline of *F*_v_/*F*_m_ for lower values of RWC_leaf_ (*e.g.*, around 20%). Fitting a logistic regression to the relationship between *F*_v_/*F*_m_ and WC_leaf_ also showed that the decrease of *F*_v_/*F*_m_ in response to WD was delayed in inoculated plants compared to non-inoculated plants and appeared for lower values of WC_leaf_ (see Figure S4 in File S1). Moreover, inoculated plants displayed higher survival probability (estimated from whole-rosette *F*_v_/*F*_m_ values; Figure 1B) at very low RWC_leaf_ (*e.g.*, around 20%), and the decline of survival as a function of RWC_leaf_ was delayed in inoculated plants compared to non-inoculated plants (Figure 5). Together these results showed that STM 196 induced a higher plant survival during stress through a good maintenance of photosynthetic efficiency at worst leaf dehydration levels.

### STM196 improves growth recovery of surviving plants, and increases biomass production

**Figure 6:**
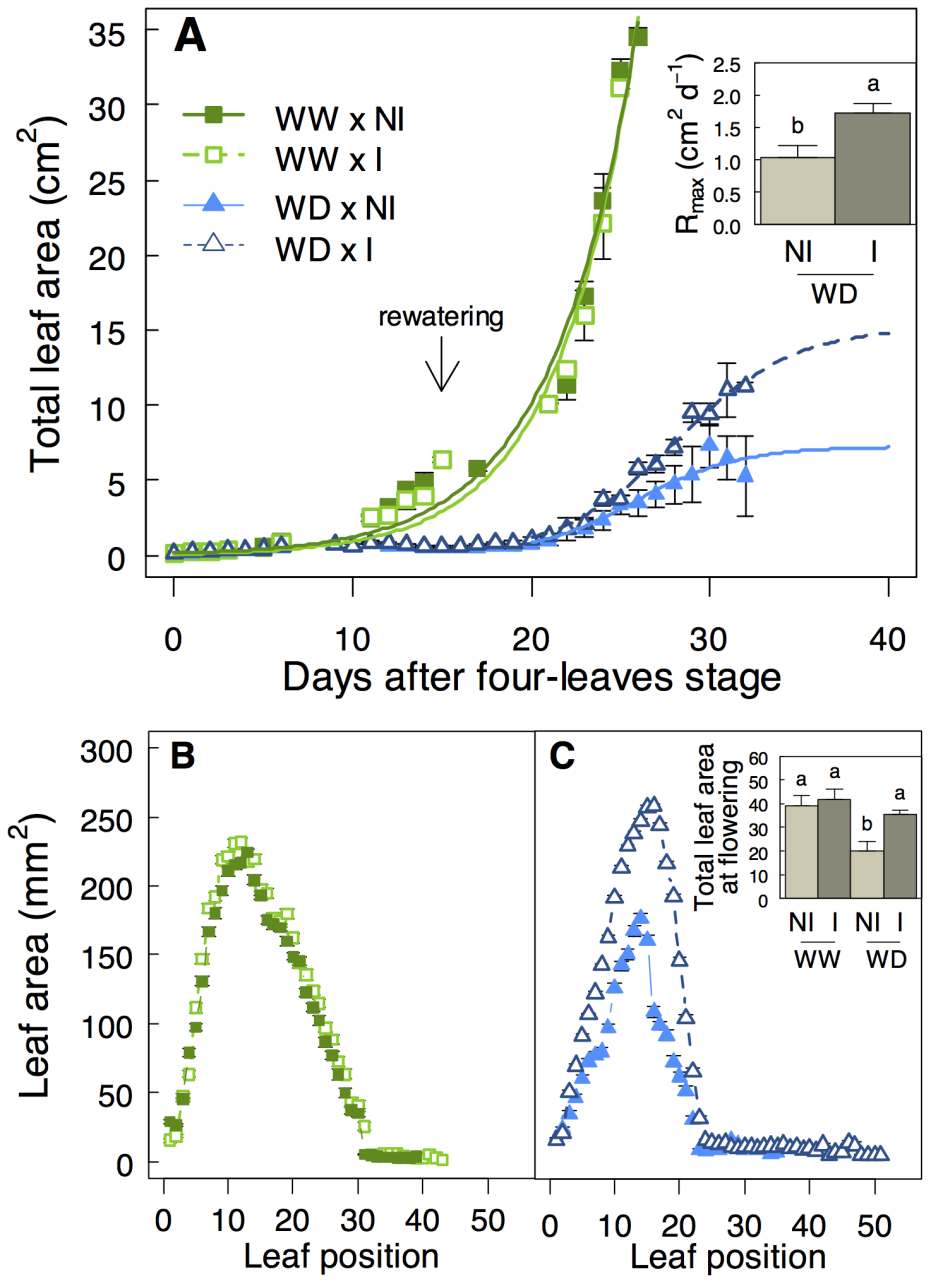
*P. brassicacearum* STM196 increases growth rate of surviving plants after rewatering. **A)** Total projected leaf area of non-inoculated (NI; closed symbols) and STM196-inoculated (I; open symbols) plants under well-watered condition (WW) and severe water deficit (WD; 6%_p_ in Figure 1) as a function of days after four-leaves stage. Arrow in A indicate the beginning of soil rewatering. Insert in **A** represents the maximum rate of leaf expansion (R_max_) after rewatering of surviving stressed plants. Area of individual leaves of I and NI plants under **B)** WW condition and **C)** WD. Insert in **C** shows total leaf area of surviving plants at flowering. Data are means (±SE) of 11–27 plants. Different letters indicate significant differences between means following Kruskal-Wallis tests (*P*<0.05).

Establishment of WD (6%_p_ stress) resulted in reduced leaf growth, and total leaf area declined until rewatering compared to plants under WW conditions (Figure 6A). Upon rewatering, leaf growth of stressed surviving plants resumed and the plants reached the reproductive stage. At flowering, WD induced a decrease by 50% of total leaf area in non-inoculated plants (insert in Fig 6C).

The plant growth promotion effect of STM196 was not effective under WW conditions but strongly occurred under WD (Figure 6). Under WD, soil inoculation by STM196 induced a significant increase in the maximum rate of leaf expansion after rewatering (R_max_; insert in Figure 6A; *P*<0.01) that led to a larger total leaf area at flowering (insert in Figure 6C; *P*<0.01). This was associated with a significant 45% increase of shoot dry biomass in inoculated plants under WD (Figure S5A in File S1). The increase in total leaf area of inoculated plants under WD was associated with larger individual leaves than non-inoculated plants (Figure 6C). At flowering, inoculated plants displayed also a higher number of leaves (Figure 6C). Flowering time was delayed by 15 d under WD but it was not affected by inoculation (Figure S5B in File S1). At flowering, all surviving plants recovered a complete rehydration of tissues (Figure S5C in File S1). Taken together, all traits demonstrated a better tolerance of inoculated plants to severe WD and an improvement of biomass accumulation upon rewatering.

## Discussion

Severe water stress induces dehydration of plant tissues and can cause irreversible cellular damages leading to death [17]. Nonetheless, plants are able to some extent to withstand periods in a dried status and restart their metabolic functions after rehydration (*e.g.*, [25], [51], [52]). Several genes in Arabidopsis have been shown to be implicated in plant survival to water deficit and transgenic modifications could improve plant survival [53]. In addition, some soil bacteria such as PGPR strains can improve tolerance to water deficit, but reports on their effects on plant survival are scarce [54], [55], specifically in response to severe water stress.

We recently showed that the PGPR *Phyllobacterium brassicacearum* strain STM196, previously isolated from the rhizosphere of oilseed rape *Brassica napus* [37], [38], improved Arabidopsis resistance to moderate water deficit through delayed developmental transitions and modifications of plant physiology, notably by a decrease of leaf transpiration through an increase of leaf abscisic acid (ABA) content [29]. Here, we show that inoculation by STM196 strain consistently induces a significant increase in survival rate under multiple scenarios of severe water deficit. We highlight that STM196 delayed and reduced mortality rate during water stress establishment through a better tolerance to leaf dehydration and leaf photosynthetic damages. Contrary to common findings where rhizobacteria enhance physiological plant status (*e.g.*, leaf water content or photosynthetic performance), here we show that STM196-inoculated plants can survive under stress with more leaf damages. Importantly, STM196 not only increased plant survival but also increased growth recovery in surviving plants and led to a higher biomass production at flowering.

### Inoculation by STM196 allows a better tolerance to leaf damages and conservation of leaf water content during stress, and a better growth recovery after rewatering

Although some studies detailed the mechanisms underlying the improvement of plant resistance to water stress by PGPR inoculation, a very few studies have showed that PGPR could improve plant survival under drastic conditions. It has been shown that some rhizobacteria, genetically modified to overproduce trehalose in their cells, can improve survival of plants under severe water-limiting conditions, notably by increasing leaf water content or by inducing the accumulation of trehalose content in the plant [54], [55]. Here, we used the automated phenotyping platform PHENOPSIS, that allows the precise control of soil watering [43], to analyze the effects of STM196, a natural PGPR, on the physiology and growth of *A. thaliana* under multiple scenarios of severe water deficit throughout the whole plant cycle. The scenarios of water deficit used in this study induced a large decrease in plant survival from 60 to 83%, which is comparable to a previous report using a similar procedure (water stress/rewatering from stage 1.04) and similar intensities of soil drying [53]. Plants inoculated by STM196 strain consistently presented a higher survival rate in comparison with non-inoculated plants.

It is well established that severe water stress strongly affects plant growth, water status and causes decline of photosynthetic capacity [17], specifically through stomatal closure and leaf senescence. Dedicated measurements require a precise knowledge of the dynamics of stress establishment and are often highly time-consuming. For this reason, non-destructive measurements based on chlorophyll fluorescence imaging have been extensively used to decipher the effects of different stresses on plant physiology (*e.g.*, [56], [57], [58]) but have rarely been used at high throughput (but see [21]). In this paper, we used chlorophyll fluorescence measurements at high throughput in order to unravel the effects of rhizobacteria on the dynamic plant responses to severe water deficit. Amongst the different photosynthetic parameters existing, dark-adapted *F*_v_/*F*_m_, reflects the maximal efficiency of PSII and is therefore one of the most used parameters for measuring leaf physiological status [20]. Most often the mean *F*_v_/*F*_m_ of a photosynthetic organ or a whole-plant is used to characterize the response to a stressor (*e.g.*, [22]). Here, we first showed that the whole-rosette mean *F*_v_/*F*_m_ was related to the probability of survival to severe water deficit. The determination of a mortality threshold allowed the estimation of survival of harvested plants and thus, the discrimination between surviving and perishing plants. The mortality threshold also allowed following the variation of plant mortality during time course. Moreover, this method was necessary to decipher the effect of an exogenous treatment that induced differences in sample size. Then, we showed that improvement of plant survival by STM196-inoculation was not related to changes in mortality threshold as determined by whole-rosette *F*_v_/*F*_m_ values but was associated to differences in tolerance to WD of surviving plants. During stress, plants are able to some extent to endure leaf photosynthetic damages. Surviving inoculated plants tolerate lower values of whole-rosette mean *F*_v_/*F*_m_ just before rewatering. The “Point of no return”, the limit point that once passed a plant dies, seemed to appear for lower values of *F*_v_/*F*_m_ in inoculated plants. The large decline in mean *F*_v_/*F*_m_ during prolonged water deficit is consistently associated with exacerbated leaf senescence [59], [60]. STM196-inoculated plants could survive with a higher proportion of leaf senescence and thus, presented a higher tolerance to leaf photosynthetic damages. Therefore, inoculated plants displayed a delayed and reduced mortality rate during water stress establishment. Leaf senescence is a common way to saving resources [18]. It allows reallocation of nutrients to reproductive organs and reduces water consumption by older and less productive leaves [61]. Leaf senescence is therefore an adaptive trait that may allow plant survival under stressful conditions [61], [62]. It has been reported that some microorganisms are able to affect photosynthetic efficiency, especially by an increase of whole-rosette *F*_v_/*F*_m_. For instance, inoculation by the PGPR *Pseudomonas fluorescens* Aur6 strain in *P. halepensis* increased mean *F*_v_/*F*_m_ value and lead to the improvement of tree growth under well-watered conditions [33]. The increase in chlorophyll content could participate to the PGPR-triggered improvement of plant photosynthetic performance [31]. Under water stress, a positive correlation between tolerance to water deficit and maintenance of PSII efficiency has been observed in rice inoculated by an arbuscular mycorrhizal fungus [63]. By contrast, it has been recently shown that inoculation by the PGPR *Bukholderia phytofirmans* PsJn strain induces a higher number of senescent leaves in *A. thaliana* at flowering under well watered conditions [64]. Here in accordance with this finding, we found that plants inoculated by PGPR could survive with more critical physiological status.

The improvement of tolerance to leaf damages by STM196-inoculation could be related to a delayed dehydration of tissues and an improved tolerance to low water status. PSII efficiency and leaf relative water content were tightly related, as previously reported by Woo et al., [22]. Traits related to leaf water status are often measured in response to rhizobacteria and drought. In response to PGPR-inoculation, it is widely accepted that rhizobacteria increase leaf water content that leads to increase plant resistance under water deprivation (*e.g.*, [65], [66], [67]). Here, inoculation by STM196 led to delayed leaf dehydration and then, at the maximum of stress severity, inoculated plants displayed a higher tolerance to low water status. Contrary to common findings, we show that STM196-inoculated plants were more likely to survive at very low water status compared to non-inoculated plants. Moreover, during water-stress establishment, STM196-inoculated plants displayed a lower decline of *F*_v_/*F*_m_ for a given leaf water content, and non-inoculated plants began to die at lower soil humidity compared to non-inoculated plants. Delayed leaf dehydration induced by STM196-inoculation could explain the delayed mortality. Dehydration delay and dehydration-tolerance are important in survival strategy [52]. These involve traits that increase access to water and decrease water losses and could result from osmolytes accumulation [68], [69], changes in stomatal conductance [70] and a large and deep root system [71]. It has been reported that inoculation by *Bacillus spp.* could alleviate negative effects of drought by affecting osmo-regulation through increasing osmoprotectors such as proline, sugars and free amino acids [72]. In the case of STM196 strain, our previous studies under moderate water deficit have shown that inoculation improves *A. thaliana*'s strategy of water saving by a developmental slowdown, a two-fold increase in root biomass and a significant decrease of transpiration rate related to an increase of ABA concentration in the leaf [29]. ABA plays a crucial role in plant responses to water stress and is involved in water loss regulation by control of stomatal closure. Modifications in leaf ABA content by STM196-inoculation could participate to delay and improve tolerance to dehydration and may be a cause of a better survival of plants under severe water stress. Moreover, it has been showed that changes in ABA content could also play a crucial role in the carbon remobilization from senescing leaves of drought-stressed plants [61]. Some other bacteria have also the capacity to modulate ABA metabolism in plants. For instance, recent work showed that inoculation by *Bacillus licheniformis* induces delayed water losses in grapevine that was correlated to an increase of ABA in leaf tissues [73]. Inoculation by STM196 may allow plants to be more efficient to cope with water scarcity in soils.

After rewatering, plant processes such as photosynthesis [23], transpiration [74], plant water status and growth [25] progressively recover their potential. Leaf growth rate followed the variation of soil water availability, and thus its decrease occurred progressively during water stress establishment. Upon rewatering, surviving plants resumed their growth and developed new leaves. We showed that inoculation by STM196 induced a better growth rate after rewatering and led to a large increase in biomass at flowering. Inoculated plants reached a similar biomass at flowering than non-stressed plants. This is due to an increase in both the number and size of leaves. This result was in accordance with our previous findings under moderate water deficit [29], where inoculation by STM196 allowed a 2-fold increase in plant biomass related to an increase in number and size of individual leaves. However, contrary to the findings under moderate water deficit, improvement of plant biomass by STM196 was not related to a delayed flowering time after rewatering.

STM196 may therefore allow a better conservation of leaf water content during stress establishment and help maintaining physiological integrity in a dried state, and then a better growth recovery when soil conditions become suitable for plant growth. The underlying physiological and molecular processes that could be involved in cells viability and growth potential remain to be elucidated.

## Conclusion

Overall our findings indicate that inoculation by *Phyllobacterium brassicaceraum* STM196 strain reinforced the survival strategy of *A. thaliana* under conditions of severe water stress. STM196 induced a better tolerance to leaf damages through delayed leaf dehydration during water stress establishment that could allow a better conservation of cell integrity and thus, growth recovery when soil conditions became favorable again. Remarkably, STM196 allowed a production of plant biomass similar to non-stressed plants. Improvement of plant tolerance to water stress is a real challenge for crop breeding, especially under global climate change. The use of plant-bacteria interactions to enhance plant tolerance to abiotic stresses in the field offers valuable and promising prospects in addition or in complement to the classical strategies of genetic selection.

## Supporting Information

File S1 **Supporting information.** Table S1, Soil chemical properties of the compost (Neuhaus N2), soil and two mixtures of both. Mixture 1 was sampled before experimentation and mixture 2 was sampled after experimentations. nd: not determined. Soil analysis was performed by ALFA Agricultural Service and Research Building, Soil Testing Laboratory of Auburn University. Figure S1, Soil water potential during soil drying. Soil water potential was determined using a potentiometer (WP4-T dewpoint meter, Decagon Devices, Pullman, WA 99163, USA) during soil drying (from 0.35 to 0.06 g H_2_O g^−1^ dry soil). Figure S2, Growth of *P. brassicacearum* STM196 strain is not affected by soil water deficit. Growth of STM196 strain was represented by cfu/mg of soil under well-watered condition (WW) and water deficit (WD). Data are means (±SE) of 3 replicates. Figure S3, Whole-rosette mean *F*_v_/*F*_m_ is not affected by inoculation under well watered condition (WW). Mean *F*_v_/*F*_m_ of non-inoculated plants (NI; closed squares) and inoculated plants (I; open squares) during time courses. Data are means (±SE) of 3–32 plants. Grey points represent individuals for each condition (NI; closed symbols and I; open symbols). Figure S4, *P. brassicacearum* STM196 induces a delayed decrease of *F*_v_/*F*_m_ in response to WD. Relationship between whole-rosette *F*_v_/*F*_m_ and leaf relative water content of non-inoculated (NI; closed symbols) and STM196-inoculated (I; open symbols) plants under well watered (WW) and water deficit (WD; 6%_p_) during soil drying (35%, 20%, 10% and 6%) and after rewatering (20%r, 35%r and 35%r at flowering). The dashed line represents the 90%-mortality threshold. Surviving plants with mean *F*_v_/*F*_m_ values above the threshold, are represented by triangles (n = 3–10 and n = 3–19 for NI and I plants, respectively) and perishing plants, with mean *F*_v_/*F*_m_ below the threshold, are represented by upside-down triangles (n = 3–9 and n = 3 for NI and I plants, respectively). Figure S5, Effect of *P. brassicacearum* STM196 strain and water deficit on growth, physiology and development of *A. thaliana* at flowering. A) Dry mass of rosette leaves, B) days to flowering and C) leaf relative water content of non-inoculated (NI) and inoculated (I) plants under well watered (WW) and severe water deficit (WD; 6%_p_). Data are means (±SE) of 11–27 plants. Different letters indicate significant differences following Kruskal-Wallis test (*P*<0.05).
(DOCX)
