# Supplementary material for: Interact to Survive: Phyllobacterium brassicacearum Improves Arabidopsis Tolerance to Severe Water Deficit and Growth Recovery
Source: PLoS One. 2014 Sep 16;9(9):e107607. doi: 10.1371/journal.pone.0107607 (PMC4166611; doi:10.1371/journal.pone.0107607)
Supplement: File S1 — Supporting information. Table S1, Soil chemical properties of the compost (Neuhaus N2), soil and two mixtures of both. Mixture 1 was sampled before experimentation and mixture 2 was sampled after experimentations. nd: not determined. Soil analysis was performed by ALFA Agricultural Service and Research Building, Soil Testing Laboratory of Auburn University. Figure S1, Soil water potential during soil drying. Soil water potential was determined using a potentiometer (WP4-T dewpoint meter, Decagon Devices, Pullman, WA 99163, USA) during soil drying (from 0.35 to 0.06 g H2O g−1 dry soil). Figure S2, Growth of P. brassicacearum STM196 strain is not affected by soil water deficit. Growth of STM196 strain was represented by cfu/mg of soil under well-watered condition (WW) and water deficit (WD). Data are means (±SE) of 3 replicates. Figure S3, Whole-rosette mean F v/F m is not affected by inoculation under well watered condition (WW). Mean F v/F m of non-inoculated plants (NI; closed squares) and inoculated plants (I; open squares) during time courses. Data are means (±SE) of 3–32 plants. Grey points represent individuals for each condition (NI; closed symbols and I; open symbols). Figure S4, P. brassicacearum STM196 induces a delayed decrease of F v/F m in response to WD. Relationship between whole-rosette F v/F m and leaf relative water content of non-inoculated (NI; closed symbols) and STM196-inoculated (I; open symbols) plants under well watered (WW) and water deficit (WD; 6%p) during soil drying (35%, 20%, 10% and 6%) and after rewatering (20%r, 35%r and 35%r at flowering). The dashed line represents the 90%-mortality threshold. Surviving plants with mean F v/F m values above the threshold, are represented by triangles (n = 3–10 and n = 3–19 for NI and I plants, respectively) and perishing plants, with mean F v/F m below the threshold, are represented by upside-down triangles (n = 3–9 and n = 3 for NI and I plants, respectively). Figure S5, Effect of P. brassicacearu [file pone.0107607.s001.docx]

# Interact to survive: *Phyllobacterium brassicacearum* improves Arabidopsis tolerance to severe water stress and growth recovery

Justine Bresson, François Vasseur, Myriam Dauzat, Marc Labadie, Fabrice Varoquaux, Bruno Touraine, Denis Vile^*^

## Supplemental Information

**Table S1. Soil chemical properties of the compost (Neuhaus N2), soil and two mixtures of both.** Mixture 1 was sampled before experimentation and mixture 2 was sampled after experimentations. nd means that the value was not determined. Soil analysis was performed by ALFA Agricultural Service and Research Building, Soil Testing Laboratory of Auburn University.

**Figure S1. Soil water potential during soil drying.** Soil water potential was determined using a potentiometer (WP4-T dewpoint meter, Decagon Devices, Pullman, WA 99163, USA) during soil drying (from 0.35 to 0.06 g H_2_O g^-1^ dry soil).

**Figure S2. Growth of *P. brassicacearum* STM196 strain is not affected by soil water deficit**. Growth of STM196 strain was represented by cfu/mg of soil under well-watered condition (WW) and water deficit (WD). Data are means (±SE) of 3 replicates.

**Figure S3. Whole-rosette mean *F*_v_/*F*_m_ is not affected by inoculation under well watered condition (WW)**. Mean *F*_v_/*F*_m_ of non-inoculated plants (NI; closed squares) and inoculated plants (I; open squares) during time courses. Data are means (±SE) of 3-32 plants. Grey points represent individuals for each condition (NI; closed symbols and I; open symbols).

**Figure S4. *P. brassicacearum* STM196 induces a delayed decrease of *F*_v_/*F*_m_ in response to WD**. Relationship between whole-rosette *F*_v_/*F*_m_ and leaf relative water content of non-inoculated (NI; closed symbols) and STM196-inoculated (I; open symbols) plants under well watered (WW) and water deficit (WD; 6%_p_) during soil drying (35%, 20%, 10% and 6%) and after rewatering (20%r, 35%r and 35%r at flowering). The dashed line represents the 90%-mortality threshold. Surviving plants with mean *F*_v_/*F*_m_ values above the threshold, are represented by triangles (n = 3-10 and n = 3-19 for NI and I plants, respectively) and perishing plants, with mean *F*_v_/*F*_m_ below the threshold, are represented by upside-down triangles (n = 3-9 and n = 3 for NI and I plants, respectively).

**Figure S5. Effect of *P. brassicacearum* STM196 strain and water deficit on growth, physiology and development of *A. thaliana* at flowering**. **A)** Dry mass of rosette leaves, **B)** days to flowering and **C)** leaf relative water content of non-inoculated (NI) and inoculated (I) plants under well watered (WW) and severe water deficit (WD; 6%_p_). Data are means (±SE) of 11-27 plants. Different letters indicate significant differences following Kruskal-Wallis test (*P* < 0.05).

**Table S1**

|  |  |  |  | **Substrats** | | | |
| --- | --- | --- | --- | --- | --- | --- | --- |
|  | **Abbreviation** | **Methods** | **Units** | **Compost N2** | **Soil** | **Mixture 1** | **Mixture 2** |
| **Calcium** | Ca | Dry Ash analysed by inductively coupled plasma (ICP) | % in soil | 1.95 | 17.21 | 17.11 | 12.69 |
| **Potassium** | K |  | % in soil | 0.16 | 0.15 | 0.20 | 0.22 |
| **Magnesium** | Mg |  | % in soil | 0.25 | 0.35 | 0.42 | 0.34 |
| **Phophorus** | P |  | % in soil | 0.08 | 0.067 | 0.093 | 0.063 |
| **Aluminum** | Al |  | ppm | 997 | 7486 | 6854 | 7934 |
| **Arsenic** | As |  | ppm | < 0.1 | < 0.1 | < 0.1 | < 0.1 |
| **Boron** | B |  | ppm | 11 | 22 | 26 | 22 |
| **Barium** | Ba |  | ppm | 183 | 732 | 746 | 770 |
| **Cadnium** | Cd |  | ppm | < 0.1 | < 0.1 | < 0.1 | < 0.1 |
| **Chromium** | Cr |  | ppm | 16 | 18 | 28 | 29 |
| **Copper** | Cu |  | ppm | 14 | 33 | 21 | 23 |
| **Iron** | Fe |  | ppm | 835 | 5123 | 6353 | 7070 |
| **Manganese** | Mn |  | ppm | 81 | 361 | 466 | 467 |
| **Molybdenum** | Mo |  | ppm | < 0.1 | < 0.1 | < 0.1 | < 0.1 |
| **Sodium** | Na |  | ppm | 2029 | 766 | 1483 | 1480 |
| **Nickel** | Ni |  | ppm | 78 | 518 | 551 | 760 |
| **Lead** | Pb |  | ppm | < 0.1 | < 0.1 | < 0.1 | < 0.1 |
| **Zinc** | Zn |  | ppm | 31 | 46 | 45 | 43 |
| **Ammonium-Nitrogen** | NH4-N | Saturated paste extraction analyzed | ppm | 0.2 | 0.1 | 0.2 | 0.3 |
| **Nitrate-Nitrogen** | NO3-N |  | ppm | 114 | 56 | 216 | 428 |
| **Nitrogen** | N | Dry combustion | % in soil | 1.10 | 0.11 | 0.18 | 0.24 |
| **Carbon** | C |  | % in soil | 40.36 | 7.27 | 10.48 | 9.75 |
| **Electrical Conductivity** | EC | Solubridge | mmhos/cm | 2.7 | 1.2 | 2.5 | 5.1 |
| **Soluble Salts** | SS |  | ppm | 1910 | 868 | 1736 | 3559 |

**Figure S1**

**Figure S2**

**Figure S3**

**Figure S4**

**Figure S5**
